# Di-(2-Ethylhexyl) Phthalate Metabolites in Urine Show Age-Related Changes and Associations with Adiposity and Parameters of Insulin Sensitivity in Childhood

**DOI:** 10.1371/journal.pone.0117831

**Published:** 2015-02-23

**Authors:** Arianna Smerieri, Chiara Testa, Pietro Lazzeroni, Francesca Nuti, Enzo Grossi, Silvia Cesari, Luisa Montanini, Giuseppe Latini, Sergio Bernasconi, Anna Maria Papini, Maria E. Street

**Affiliations:** 1 Department of Pediatrics, University Hospital of Parma, Parma, Italy; 2 Interdepartimental Laboratory of Peptide & Protein Chemistry & Biology (www.peptlab.eu), Florence, Italy; 3 Department of Chemistry “Ugo Schiff,” University of Florence, Sesto Fiorentino, Italy; 4 PeptLab@UCP c/o LCB EA 4505 Université de Cergy-Pontoise, Cergy-Pontoise, France; 5 Villa Santa Maria Institute, Tavernerio, Italy; 6 Clinical Physiology Institute (IFC-CNR), National Research Council of Italy, Lecce Section, Lecce, Italy; 7 Division of neonatology, Perrino Hospital, Brindisi, Italy; 8 Depts of Paediatrics and of Research and Statistics, S. Maria Nuova Hospital and Research Institute, Reggio Emilia, Italy; Qingdao Agricultural University, CHINA

## Abstract

**Objectives:**

Phthalates might be implicated with obesity and insulin sensitivity. We evaluated the levels of primary and secondary metabolites of Di-(2-ethylhexyl) phthalate (DEHP) in urine in obese and normal-weight subjects both before and during puberty, and investigated their relationships with auxological parameters and indexes of insulin sensitivity.

**Design and Methods:**

DEHP metabolites (MEHP, 6-OH-MEHP, 5-oxo-MEHP, 5-OH-MEHP, and 5-CX-MEHP), were measured in urine by RP-HPLC-ESI-MS. Traditional statistical analysis and a data mining analysis using the Auto-CM analysis were able to offer an insight into the complex biological connections between the studied variables.

**Results:**

The data showed changes in DEHP metabolites in urine related with obesity, puberty, and presence of insulin resistance. Changes in urine metabolites were related with age, height and weight, waist circumference and waist to height ratio, thus to fat distribution. In addition, clear relationships in both obese and normal-weight subjects were detected among MEHP, its products of oxidation and measurements of insulin sensitivity.

**Conclusion:**

It remains to be elucidated whether exposure to phthalates *per se* is actually the risk factor or if the ability of the body to metabolize phthalates is actually the key point. Further studies that span from conception to elderly subjects besides further understanding of DEHP metabolism are warranted to clarify these aspects.

## Introduction

Obesity, insulin resistance, and type 2 diabetes are interrelated metabolic disorders which prevalence has increased substantially in the past two decades [1]. Insulin resistance occurs when increasing amounts of insulin are required to correctly regulate transport of plasma glucose into peripheral tissues. Although the precise mechanism is unclear, insulin resistance is commonly associated with an increase in central (visceral) obesity [2].

Some studies have reported an association between persistent exposure to some organic pollutants, such as phthalates, bisphenol A, heavy metals and polychlorobiphenyl, and increased body weight and diabetes [3], antagonism to the action of thyroid hormone nuclear receptors with a reduction in fT3 and fT4 [4,5] and an increase in oxidative stress [6]. Oxidative stress is related with inflammation and inflammation itself is a cause of insulin resistance [7]. Obesity is characterised also by a low grade of chronic inflammation [8], and in addition mechanisms inducing insulin resistance, mediated by pro-inflammatory cytokines have been described [7,9].

High molecular weight phthalates, such as Di-(2-ethylhexyl) phthalate (DEHP), are primarily used as plasticizers for the manufacturing of polyvinyl chloride (PVC), which is used extensively in consumer products, flooring and wall coverings, as well as food contact applications, medical devices, toys and cosmetic containers [10]. In the United States more than 75% of the population has measurable levels of urinary metabolites of phthalates [11].

A causal role for high-molecular-weight phthalates in obesity is biologically plausible [12–16]. Perturbation of thyroid function has also been considered a possible cause for increasing body mass index (BMI) [17], and some authors claim that their anti-androgenic effect could also be implicated with increasing BMI [18].

Concentrations of urinary phthalate metabolites have been found to be associated with increased waist circumference and insulin resistance in adult males [2,19].

Phthalates are rapidly metabolized in the body, with elimination half-lives of less than 24 hours [20]. Selected phthalate monoesters, such as mono(2-ethylhexyl)phthalate (MEHP) are also reproductive and developmental toxicants [21].

Urine is the preferred matrix for phthalate determination in humans [22]. Because of rapid metabolism urinary metabolite levels are typically higher, and therefore, more precisely measured than levels of the parent compound found in other media. Several studies have examined the within-person variability of phthalate metabolites and concluded that despite their short half-lives, exposure may be sufficiently stable to assign an exposure level based on a single sample [23–25].

In recent years, systems biology approaches have developed. These methods applied to biomedical and biological data attempt to model and discover specific properties and complex interactions within biological systems. These comprise artificial adaptive systems (AAS) which use is rapidly spreading in biology and medicine [26] besides other disciplines, and could represent an excellent method to study the effects of environmental contaminants on human biology. Auto Contractive Map (Auto-CM) a new Artificial Neural Network [27] is effective at highlighting any kind of consistent pattern and/or systematic relationships, and hidden trends and associations among variables, in particular is able to describe a context which is typical of living systems where a continuous time dependent complex change in the variable value is present.

We hypothesized that exposure to phthalates was related with obesity and insulin resistance, and we aimed to evaluate the levels of the primary and secondary metabolites of DEHP in urine in obese and normal-weight subjects both before and during puberty, and to investigate their relationships with auxological parameters and indexes of insulin sensitivity using both traditional statistical analysis (i.e.Mann-Whitney test, Kruskall-Wallis ANOVA, correlation tests) and Auto-CM analysis.

## Materials and Methods

### Subjects and auxological observations

**Fig 1:**
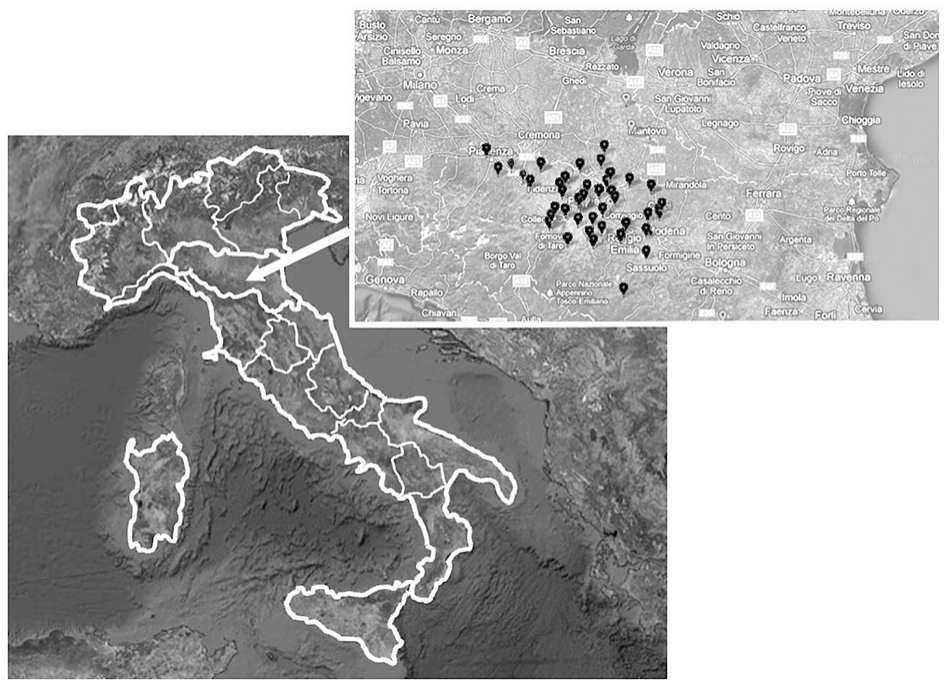
Geographical distribution of the obese and normal-weight subjects. The children came from a small area within the Emilia-Romagna region in Italy, mainly between the provinces of Parma and Reggio-Emilia.

Both prepubertal and pubertal obese (N:41) and normal-weight children (N:31) were enrolled consecutively at the paediatric endocrine clinic at the University Hospital in Parma. The children were comparable for age, sex and pubertal stages. The features of these subjects are summarized in Table 1. All subjects came from the same geographical area where they were born (Fig. 1). Controls were subjects having normal familial short stature, negative endocrine examinations, no chronic diseases and negative celiac screening. For the obese subjects, endocrine disorders, chronic diseases and genetic syndromes or dysmorphic features were considered exclusion criteria. Height (Ht) and weight were measured in all subjects using a Harpenden stadiometer, and an electronic scale, respectively. Ht and mid-parental height (MPHt) were expressed as standard deviation scores (SDS) using the Italian reference data [28].

**Table 1:** Clinical characteristics, anthropometric data, and measurements of insulin sensitivity in obese and normal-weight subjects (controls).

Parameter	Controls	Obese	p
Number	31	41	
Males/Females	19/12	22/19	
Pubertal/Prepubertal	20/11	27/14	
Chronological age (yr)	12.69 ± 0.66	12.53 ± 0.48	ns
Height SDS	-0.91 ± 0.30	0.91 ± 0.16	0.0001
Mid Parental Ht SDS	-0.21 ± 0.18	-0.12 ± 0.15	ns
BMI SDS (Cole)	-0.12 (-0.88–0.75)	3.41 (3.04–3.59)	0.0001
Birth weight (Kg)	3.04 ± 0.13	3.4 ± 0.09	0.01
Waist circumference (cm)		107.0 ± 2.54	
Waist circumference/height ratio		0.68 ± 0.01	
Chronological age at onset of obesity (yr)		5.2 ± 0.50	
Insulin (IU/L)	8.5 ± 1.2	15.9 ± 1.9	0.002
Glucose (mg/dl)	80.2 ± 1.2	82.8 ± 1	ns
FGIR	13.2 (6.67–20.0)	6.4 (4.42–9.44)	0.001
HOMA-IR	1.09 (0.79–2.56)	2.46 (1.88–3.43)	0.0001
WBISI		3.64 ± 0.25	
AUCG		234.8 (214–261)	
AUCI		225.5 ± 17.0	

Data are mean± SEM for normal distribution, median (25^th^-75^th^ percentile) for asymmetric distribution. FGIR: Fasting glucose to insulin ratio; WBISI: Whole Body Insulin Sensitivity Index; AUCG: Area under the curve for glucose calculated from the Oral Glucose Tolerance Test (OGTT); AUCI: Area under the curve for insulin calculated from the OGTT.

Body mass index (BMI) was calculated as weight/height^2^ (Kg/m2), and standardized according Cole’s reference data [29]. Subjects with a BMI above the one indicated for age and sex according to the IOFT criteria [30] were considered obese. These were also all above the 95th centile according to the Italian reference data, and the control subjects had normal weight (BMI < 75th centile according to Italian reference data) [28]. Waist circumference and waist to height ratio were taken in addition in the obese subjects to assess fat distribution [31].

Based on breast development or genitalia staging subjects were classified in pubertal and prepubertal according to Tanner’s criteria [32,33].

### Measurements of insulin sensitivity

All obese subjects underwent an oral glucose tolerance test (OGTT) after an overnight fast. A glucose load of 1.75 g/kg body weight (max 75 g) in 300 mL water was prepared and administered in 3 min. Blood samples were taken prior to, and 30, 60, 90 and 120 min after glucose ingestion. In all subjects insulin and glucose were assessed at all time points.

The insulinogenic index was calculated as Δ insulin (0–30 min) divided by Δ glucose (0–30 min) where the values were calculated at baseline and at 30 min during OGTT, and was used as an index of pancreatic β-cell function [35].

The fasting glucose-to-insulin ratio (FGIR) was calculated as the ratio of fasting plasma glucose (G_0_) divided by fasting plasma insulin (I_0_) levels [35] where G_0_ was in milligrams per deciliter and I_0_ in microunits per milliliter.

Homeostasis model assessment estimate of insulin resistance (HOMA-IR) was calculated, according to the following formula [34]: $\frac{G_{0}\times I_{0}}{22.5}$where G_0_ and I_0_ were basal fasting glucose and insulin, respectively. Glucose was expressed in mmol/L and insulin in mIU/mL. HOMA-IR indexes were classified according to criteria for age and Tanner stage of puberty [36].

The whole body insulin sensitivity index (WBISI) was calculated based on insulin (IU/mL) and glucose (mg/dl) concentrations obtained during the OGTT and the corresponding fasting values, as originally described by Matsuda and DeFronzo [35]. Areas under the curve for insulin (AUCI) and glucose (AUCG), were calculated using trapezoidal integration [37].

The presence of metabolic syndrome was established based on the International Diabetes Federation criteria [38].

Insulin sensitivity parameters of obese and normal-weight subjects are reported in Table 1.

Plasma glucose concentrations were assayed using a polarographic method (Synchron CX systems).

Insulin concentrations were measured using a chemiluminescence method by Diagnostic Products Corporation (Los Angeles, CA, USA) for reading by Immulite2000. The intra-assay CV was 6.5, the inter-assay CV 7.1%.

### Determination of DEHP secondary metabolites in urine

First morning urine specimens were collected in all subjects, and stored at -20°C until assayed.

**Fig 2:**
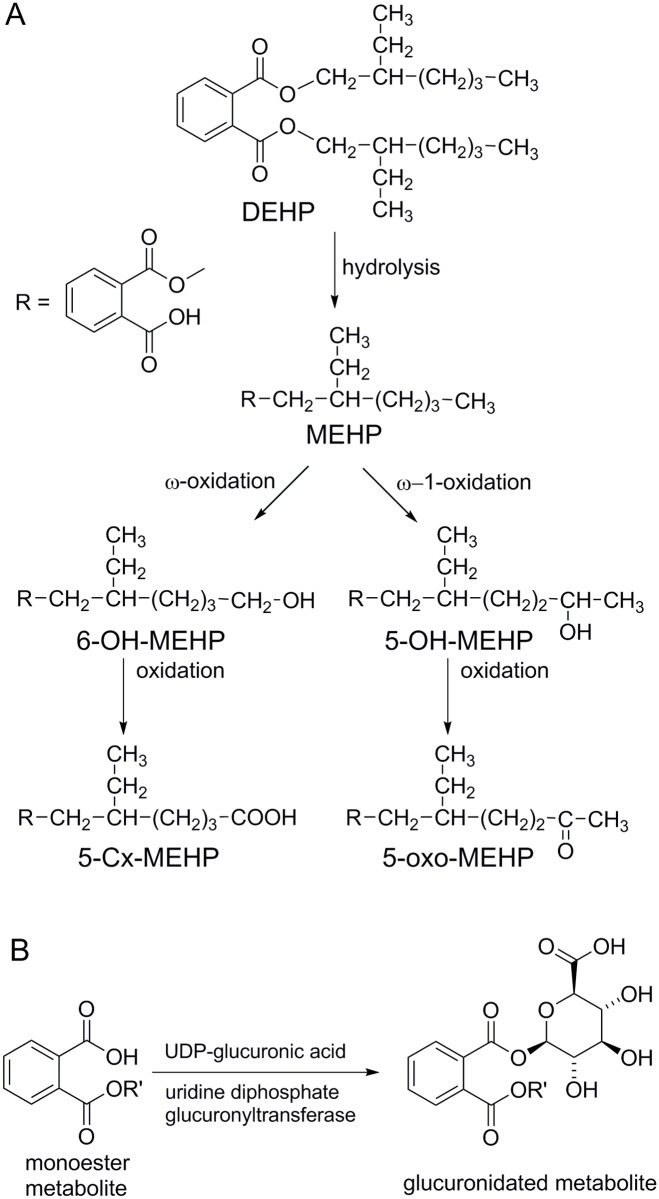
Metabolism of DEHP. (A) Hydrolytic/oxidative pathway leading to MEHP and secondary metabolite formation. (B) Formation of glucuronic conjugates of DEHP metabolites.

The following metabolites were measured as previously described [39]: MEHP (mono-(2-ethylhexenyl) 1,2-benzenedicarboxylate) and 6-OH-MEHP (mono-(2-ethyl-6-hydroxyhexyl) 1,2-benzenedicarboxylate), 5-oxo-MEHP(mono-(2-ethyl-5-oxohexyl) 1,2-benzenedicarboxylate), 5-Cx-MEHP (mono-(2-ethyl-5-carboxypentyl) 1,2-benzenedicarboxylate, and 5-OH-MEHP (mono-(2-ethyl-5-hydroxyhexyl) 1,2-benzenedicarboxylate) (Fig. 2). Each single metabolite, used as pure analytical standard (<98% purity), was synthesized in the Laboratory of Peptide and Protein Chemistry and Biology (PeptLab) at the University of Florence, following the previously described procedure [40]. All the solvents, labware and instrumentation used during the solid phase extraction (SPE) procedure and the HPLC-ESI-MS analytical process, were verified to be MEHP and secondary oxidative metabolites free.

DEHP metabolite concentrations in each single specimen were normalized according to urinary creatinine concentration. All children had urine creatinine within the normal reference range for age. Urine creatinine measurements were assesed using a Synchron AS/ASTRA clinical analyser (Beckman Instruments). We used values (μg/L) for creatinine and (g/L) for dilution correction in the analyses, therefore the concentration of each metabolite in urine was expressed as μg/g.

Human urine samples (1 mL) were defrosted, sonicated, mixed and dispensed in glass tubes prior to assaying. Then ammonium acetate buffer (250 mL, pH 6.5) was added. Incubation with β glucuronidase (5 mL, 200 units/mL, Roche Biochemical) was performed at 37°C for 90 min, resulting in quantitative glucuronide hydrolysis of phthalates and metabolites. After deconjugation, samples were treated with two steps of SPE, (SPE cartridges 3 mL/60 mg of Oasis HBL, Waters) to remove any contamination of biological matrix, following the procedure described by Blount et al. [41]. Analytes were finally eluted with acetonitrile and ethyl acetate, concentrated, re-suspended in water. All the samples were analysed by RP-HPLC-ESI-MS. One blank and one quality-control (QC) sample were included in each batch of samples. The QC sample was spiked with pooled urine, and MEHP and secondary oxidative metabolite standards in known concentration (200 ng/mL). The lower limits of quantification (LOQs) were 0.042 μg/L MEHP, 0.048 μg/L 5-OH-MEHP, 0.049 μg/L 5-oxo-MEHP, 0.0051 5-Cx-MEHP and 0.008 μg/L 6-OH-MEHP. In urine, the limits of detection (LODs) were 0.014 μg/L MEHP, 0.016 μg/L 5-OH-MEHP, 0.016 μg/L 5-oxo-MEHP, 0.019 5-Cx-MEHP and 0.002 μg/L 6-OH-MEHP. Calibration curves for the quantitative urine analysis were calculated for all analytes plotting peak area average (y) against concentration of standards (x). Five standard solutions (linear range: 2.5–2500 ng/mL) for calibration curve plotting, were prepared for all the metabolites. Curves with correlation coefficients (r^2^) greater than 0.998 were generated (MEHP 0.999, 5-OH-MEHP 0.999, 5-oxo-MEHP 0.999, 5-Cx-MEHP 0.999 and 6-OH-MEHP 0.998).

### Ethical Committee Approval

Informed consent was obtained from the parents of subjects and controls as appropriate. The study was approved by the local Ethics Committee in Parma.

### Statistical Analysis

Statistical analysis was carried out using the SPSS package 18.0. Any normal distribution of data was assessed by the Kolmogorov-Smirnov test. As no significant difference was detected in this series between males and females, subjects of both sexes were analyzed together.

To analyze differences between obese and normal-weight subjects, and differences between subgroups of obese subjects we used Student’s T test for unpaired data or Mann-Whitney test, and Kruskall-Wallis ANOVA followed by Bonferroni’s correction as appropriate. Non-parametric Spearman correlation test was used to measure associations between variables and DEHP metabolites. A multiple linear regression analysis was performed to establish which were the major determinants of MEHP urinary concentration.

Data are expressed as mean±SEM, and as median and 25^th^-75^th^ percentiles as appropriate. Only significant correlations are reported in the text.

### Auto Semantic Connectivity Map (Auto-CM) analysis

Auto-CM is a new data mining tool based on an Artificial Neural Network that is effective at highlighting any kind of consistent pattern and/or systematic relationships, and hidden trends and associations among variables. The architecture and mathematics of Auto-CM were described elsewhere [27]. Briefly, Auto-CMs‘spatializes’ the correlation among variables by building a suitable embedding space where a visually transparent and cognitively natural notion such as ‘closeness’ among variables reflects accurately their associations. Auto-CM converts this ‘closeness’ into a compelling graph-theoretical representation that picks all and only the relevant correlations and organizes them into a coherent picture. Moreover, it fully exploits the topological meaning of graph-theoretical representations in that actual paths connecting vertices (variables) in the representation carry a definite meaning in terms of logical interdependence in explaining the data set’s variability. The Auto-CM is characterized by a three-layer architecture: an Input layer, where the signal is captured from the environment, a Hidden layer, where the signal is modulated inside the Auto-CM, and an Output layer, through which the AutoCM feeds back upon the environment on the basis of the stimuli previously received and processed.

The input variables for each model were: MEHP, 6-OH-MEHP, 5-oxo-MEHP, 5-OH-MEHP, 5-Cx-MEHP, FGIR, HOMA index, insulinogenic index, HtSDS, BMISDS, and birth weight SDS. Quite uncommonly, the weights determined by Auto-CM after the training phase, admit a direct interpretation. Specifically, they are proportional to the strength of many-to-many associations across all variables. Subsequently, association strengths are visualized by transforming weights into physical distances: i.e. couples of variables which connection weights are higher get relatively nearer and viceversa if connection weights are lower, they get further apart. By applying a simple mathematical filter such as the minimum spanning tree to the matrix of distances, a graph is generated [42,43], termed connectivity map [27]. This representation allows a visual mapping of the complex web of connection schemes among variables, and greatly eases the detection of the variables that play a key role in the schemes. The system provides also a quantification of the ‘strength’ of links among variables (nodes of the graph) by a numerical coefficient. The strength of the link ranges from 0 (minimum strength) to 1 (maximal strength). In this study we transformed 11 input continuous variables in 22 input variables constructing for each of them, scaled from 0 to 1, its complement. In the map we have named these two different forms as high and low [44].

## Results

### Differences in DEHP metabolite concentrations

**Urinary DEHP metabolites in obese children and controls.** The percentages of detectable DEHP metabolites in urine samples from obese and normal-weight (control subjects) are reported in Table 2. 5-Cx-MEHP and 6-OH-MEHP were less detectable in normal-weight than in obese subjects.

**Table 2:** DEHP metabolites were measurable in the urine samples of obese and normal-weight (control subjects) in the following percentages with respect to the total number of subjects enrolled in each of the two groups.

	MEHP	5CX-MEHP	5OH-MEHP	5OXO-MEHP	6OH-MEHP
OBESE	75.6%	80.5%	80.5%	87.8%	87.8%
CONTROLS	96.8%	38.7%	83.9%	74.2%	51.6%

**Fig 3:**
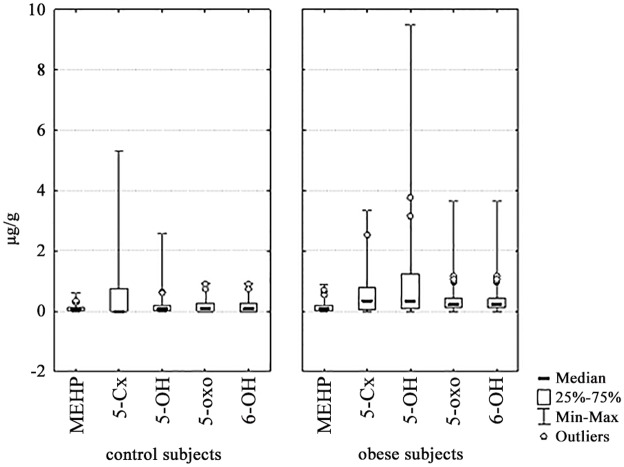
Concentration and distribution of single DEHP metabolites in urine. (A) control subject and (B) obese children and adolescents.

Obese children had increased 5-OH-MEHP, 5-oxo-MEHP, and 6-OH-MEHP concentrations, compared with normal-weight children, as reported in Fig. 3.

Both obese and control subjects were subsequently subdivided according to presence or absence of puberty (Table 3).

**Table 3:** Concentration of DEHP metabolites in urine normalized by creatinine (espressed as μg/g).

	A Controls Prepubertal	B Controls Pubertal	C Obese Prepubertal	D Obese Pubertal	p	Significant differences
MEHP	0.15 (0.05–0.34)	0.04 (0.02–0.08)	0.27 (0.08–0.44)	0.1 (0.03–0.18)	0.004	A v sB;A vs C;B vs D
5OH-MEHP	0.14 (0.08–0.53)	0.1 (0.04–0.15)	1.24 (0.27–2.67)	0.43 (0.23–1.97)	0.0001	C vs D;
5CX-MEHP	1.46 (0.49–2.84)	0.78 (0.50–1.58)	0.76 (0.11–0.93)	0.60 (0.28–0.90)	ns	
5OXO-MEHP	0.14 (0.04–0.53)	0.14 (0.06–0.27)	0.35 (0.06–0.74)	0.30 (0.18–0.40)	ns	
6OH-MEHP	0.41 (0.11–0.87)	0.25 (0.05–0.35)	1.05 (0.26–1.4)	0.56 (0.32–0.9)	0.01	C vs D; A vs C

Reported results are from Kruskall-Wallis ANOVA.

Data are median (25^th^-75^th^ percentile);

vs: versus.

In childhood, MEHP urine concentrations were higher in pre-puberty than in puberty both in control and obese subjects. Prepubertal controls had lower concentrations of 6-OH-MEHP than prepubertal obese subjects, and had less 5-OH-MEHP, 5-oxo-MEHP and 6-OH-MEHP concentrations than pubertal obese subjects.

**Urinary DEHP metabolites in obese children, based on the HOMA index.** 9/39 obese (23%) subjects had an increased HOMA index compatible with insulin resistance.

Obese children with normal HOMA index had significantly higher 5-oxo-MEHP concentrations than obese subjects with insulin resistance (0.52±0.13 vs 0.17±0.06 μg/g, P: 0.02). 6-OH-MEHP showed a trend to be lower in insulin resistance.

**Urinary DEHP metabolites in obese children, based on the presence or absence of the metabolic syndrome.** 18/39 obese subjects (46%) had the metabolic syndrome. No statistical significant difference was observed between the two groups, however, obese children with metabolic syndrome showed a trend to have lower 5-OH-MEHP, 5-oxo-MEHP, and 6-OH-MEHP urinary concentrations (data not shown).

### Correlation analysis in obese patients and in controls

**Correlation of DEHP metabolites with auxological parameters.** In controls, MEHP was negatively correlated with chronological age (rho:-0.349; p<0.03) and with the presence of puberty (rho:-0.564, p<0.001; Table 4).

**Table 4:** Relationships among DEHP metabolites concentration in urine normalized by creatinine (espressed as μg/g) in normal-weight children.

DEHP metabolite	Auxological and insulin sensitivity measurements	Spearman’s correlationcoefficient	p
	C. A.	-0.349	0.03
MEHP	Puberty	-0.564	0.001
	HtSDS	0.579	0.002
5-oxo-MEHP	BMISDS	0.441	0.015
	Insulin	0.365	0.05
6-OH-MEHP	FGIR	-4.467	0.01

5-oxo-MEHP correlated positively with both HtSDS (rho:0.579; p<0.002) and BMISDS (rho:0.441; p<0.015; Table 5).

**Table 5:** Relationships among DEHP metabolites concentrations in urine normalized by creatinine (expressed as μg/g) in obese children.

DEHP metabolite	Auxological and insulin sensitivity measurements	Spearman’s correlation coefficient	p
	C.A.	-0.349	0.034
MEHP	HtSDS	-0.342	0.038
	C.A. onset of obesity	-0.286	0.05
	Insulin (IU/L)	-0.331	0.045
	FGIR	0.341	0.039
	Insulinogenic Index	-0.338	0.041
	HOMA	-0.322	0.052
	Waist circumference	0.366	0.026
5-Cx-MEHP	Waist to height ratio	0.409	0.012
6OH-MEHP	AUCI	-0.322	0.052

C.A.: chronological age; HtSDS: height expressed as standard deviation score. BMISDS: Body mass index expressed as standard deviation score.

FGIR: Fasting glucose to insulin ratio; WBISI: Whole Body Insulin Sensitivity Index; AUCI: Area under the curve for insulin calculated from the from the Oral Glucose Tolerance Test (OGTT).

When normal-weight subjects were analysed separately based on the absence or presence of puberty, in prepubertal subjects the correlation between 5-oxo-MEHP and HtSDS was confirmed (rho:0.535; p<0.022), whereas in pubertal controls 5-Cx-MEHP was negatively correlated with birth weight (rho:-0.617; p<0.043), and 5-oxo-MEHP was positively correlated with BMISDS (rho:0.611; p<0.046).

In obese subjects, MEHP was negatively correlated with chronological age, as in controls (rho: -0.349, p< 0.034), and with waist circumference (rho:-0.342; p< 0.038). Interestingly, MEHP was correlated also negatively with the age at onset of obesity (rho: -0.286; p< 0.05; Table 4 B).

5-Cx-MEHP was positively correlated with waist circumference (rho: 0.366; p<0.026) and with the waist to height ratio (rho: 0.409; p< 0.012; Table 4 B).

In prepubertal obese children, 5-Cx-MEHP was positively correlated with the waist to hip ratio (rho: 0.39; p< 0.044), and with the waist circumference (rho: 0.432; p< 0.024), as in the entire group.

In the children who had started puberty only a relationship of 5-Cx-MEHP with the HtSDS ratio (rho: -0.593; p< 0.05) was found.

**Correlation of DEHP metabolites with measurements of insulin sensitivity.** In the entire group of controls, 6-OH-MEHP was positively correlated with insulin (rho: 0.365; p< 0.05) and negatively correlated with FGIR, as would be expected (rho: -0.467; p< 0.01; Table 4 A).

In prepubertal controls, these correlations were confirmed, and in addition 6-OH-MEHP was positively correlated with the HOMA-IR index (rho: 0.583; p< 0.01) and with the insulinogenic index (rho: 0.639; p< 0.0004).

In obese subjects relationships of MEHP and 6-OH-MEHP were found with all calculated measurements of insulin sensitivity (Table 4 B).

In obese prepubertal subjects a positive relationship between MEHP and WBISI was confirmed (rho: 0.431; p< 0.025).

No correlation among any metabolite and measurements of insulin sensitivity was detected in puberty.

### Multiple Regression Analysis

A multiple regression analysis was performed using MEHP as dependent variable, and HtSDS, BMISDS, gender, puberty, waist circumference, waist to height ratio, CA age at onset of obesity, HOMA, WBISI, AUCG, AUCI, and the insulinogenic index as independent variables in obese children. This showed a significant effect of puberty (p: 0.020), a near significant effect of CA age at onset of puberty (p:0.055), and of waist circumference (p: 0.059). In controls, HtSDS, BMISDS, gender, puberty, birth weight, HOMA and insulinogenic index were the available independent variables and a significant relationship was found for puberty (P: 0.001) and a near significant effect for gender (p: 0.051).

### Auto-CM analysis

**Fig 4:**
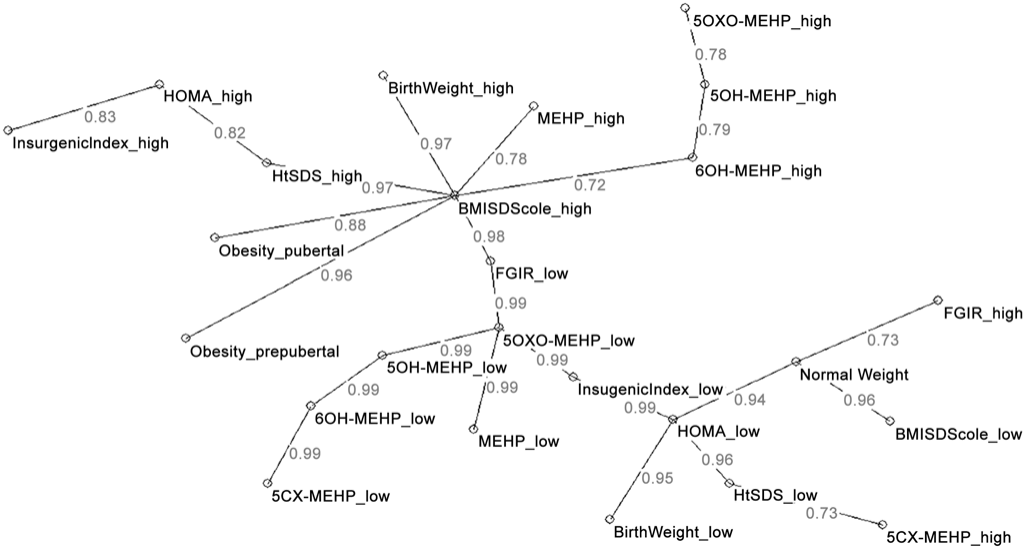
Semantic Connectivity Map linking DEHP metabolites. Analysis with auxological parameters and measurements of insulin sensitivity in normal-weight and obese children and adolescents. Values ranging from 0 (no association) to 1 (the strongest association) express the strength of association.

The main relationships among the variables emerging from the AutoCM are shown is Fig. 4.

The condition of obesity and normal-weight were clearly distinct and on opposite sides of the graph.

High MEHP and high 6-OH-MEHP concentrations were associated with a high BMISDS with a moderate-high link strength. *Vice versa* low 5-oxo-MEHP urine concentrations were associated with a low FGIR and a low insulinogenic index with a very high link strength. A high concentration of 5-Cx-MEHP was associated with a low HtSDS. Birth weight showed no relationship with the metabolites analyzed.

## Discussion

The data of this study showed changes in DEHP metabolites related with obesity, onset of puberty, and presence of insulin resistance. Changes in urine metabolites were related also with waist circumference and waist to height ratio, thus to fat distribution.

The main limitation of this study stands in the small number of subjects analysed, however, large population studies have addressed issues related to exposure but have not taken into account aspects specifically related with metabolism and very few studies have considered restricted age-groups [45]. We selected subjects all born and living in a small geographical area within the Emilia-Romagna Region in the North of Italy, an industrialized area, so that significant regional exposure differences should have been excluded. One of the major exposure routes for DEHP, one of the most diffuse phthalates, is represented, however, by food ingestion [46]. A European group estimated exposure to DEHP to be about one order of magnitude higher in children than in adults supporting the need and interest to assess exposure and metabolism in different phases of life [47]. A limitation to this study is represented by the lack of specific data relative to food and calorie intake. However, if the obese subjects had been on a diet, a restricted quality/amount of food would have also represented a limitation. We cannot rule out, however, higher food ingestion in these same subjects.

From a metabolic point of view hydrolysis of DEHP, oxidative metabolism of MEHP and organ-specific metabolites of DEHP have been previously examined in laboratory animals [48–54]. However, the hepatic and extra-hepatic metabolism of DEHP in humans has not been well characterized yet.

We detected differences between obese and normal-weight children in the concentration of DEHP metabolites in urine. In particular, 5-Cx-MEHP and 6-OH-MEHP were higher in concentration in obese than in normal-weight children. This could be generically ascribed to a different exposure and/or metabolism in the groups investigated. Omega 1 oxidation and transformation of MEHP to 5-Cx-MEHP was reduced in normal-weight subjects, whereas both ω and ω-1 oxidation end-products (Fig. 3) were increased in obesity. In prepuberty, MEHP seemed to be less oxidized, independent of obesity suggesting that metabolism physiologically changes with age but that there are differences between obese and normal-weight subjects. In the obese children, 5-oxo-MEHP was higher in the subjects having an abnormal HOMA index which could suggest that oxidation end products are increased when a degree of insulin resistance is present. Furthermore, the Auto-CM analysis showed a relationship between a low 5-oxo-MEHP concentration and a low FGIR, a feature of insulin-resistance. The low 5-oxo-MEHP concentration was also associated with a low insulinogenic index, suggestive of impaired β-cell function.

The negative relationship between MEHP and chronological age suggested an increase in MEHP metabolism with age, and onset of puberty. The negative relationship of MEHP with age at onset of obesity could suggest that a reduced capacity to metabolize MEHP might be a cause or consequence of obesity. Furthermore, we detected a negative relationship of MEHP with waist circumference, and with abdominal fat distribution (waist to height ratio) supporting a relationship between MEHP metabolism and fat distribution. The multiple regression analysis confirmed in the obese group a major effect of puberty, and an effect of chronological age at onset of obesity, and of fat distribution (waist circumference).

Moreover, when Auto-CM analysis was used, a high MEHP concentration was directly related with a high BMISDS as well as with high 6-OH-MEHP concentrations in urine. This latter finding was in accordance with other author’s findings. Wang et al. also reported this relationship in an 8–11 age-group which would have included both pre-and pubertal subjects [55], and other studies reported this same finding in much wider and heterogeneous population studies [19,25,56].

Among the downstream metabolites, in obese children, 5-Cx-MEHP was correlated with increased adipose tissue in prepuberty, whereas in puberty most relationships were lost suggesting that puberty in itself played a major role in regulating DEHP metabolism, as confirmed in the multiple regression analysis in both the normal-weight and obese children. A gender effect was seen in the normal-weight subjects but statistical significance was not attained likely due to the limited number of subjects included in this pilot study.

The positive correlation, in controls, of 6-OH-MEHP with insulin, and the HOMA index, and the negative correlation with the FGIR suggested that this metabolic pathway favoured insulin resistance. This was further confirmed by the negative relationship of 6-OH-MEHP with the amount of insulin released (AUCI) in the obese children.

In summary, these findings showed that the more MEHP was metabolised to 6-OH-MEHP, the greater the state of insulin resistance in a given subject, whereas the lesser MEHP was metabolised, the lesser insulin was required, the better the FGIR, the lower the HOMA index, and the higher the WBISI (Table 4 B).

At variance with our study, previous studies in white elderly subjects, evaluating MEHP in serum, failed to detected any relationship with the HOMA-IR index, however, this could be due both to the age-group considered and to the substrate used to determine its concentration [57, 58]. Interestingly, in our series, 6-OH-MEHP was also the only metabolite capable of discriminating between the conditions of obesity and normal- weight.

Finally, in prepubertal controls, 5-Cx-MEHP was found to be negatively correlated with birth weight. Birth weight was normal in all the children enrolled in the study, however, this might suggest a possible programming of MEHP metabolism in utero which extends at least to prepubertal years. However, the AutoCM analysis failed to find a link of birth weight with any of the metabolites studied in this study. Other authors have described relationships of other phthalates with birth weight [59], therefore, it might be that in this study we were not looking at the phthalate metabolites significantly influencing growth and weight in utero.

Possible limitations of this study stand in the sample size, in the lack of details related with food, and daily calorie intake. Furthermore, it is unknown whether the shorter stature in the controls could have affected the results.

In conclusion, metabolism changed with age, height, weight, and mostly puberty. Fat distribution and measurements of insulin sensitivity showed relationships with specific DEHP metabolites.
